# A Multi-Functional Imaging Approach to High-Content Protein Interaction Screening

**DOI:** 10.1371/journal.pone.0033231

**Published:** 2012-04-10

**Authors:** Daniel R. Matthews, Gilbert O. Fruhwirth, Gregory Weitsman, Leo M. Carlin, Enyinnaya Ofo, Melanie Keppler, Paul R. Barber, Iain D. C. Tullis, Borivoj Vojnovic, Tony Ng, Simon M. Ameer-Beg

**Affiliations:** 1 Division of Cancer Studies, Randall Division of Cell and Molecular Biophysics, King’s College London, London, United Kingdom; 2 Gray Institute for Radiation Oncology and Biology, Department of Oncology, University of Oxford, Oxford, United Kingdom; Aligarh Muslim University, India

## Abstract

Functional imaging can provide a level of quantification that is not possible in what might be termed traditional high-content screening. This is due to the fact that the current state-of-the-art high-content screening systems take the approach of scaling-up single cell assays, and are therefore based on essentially pictorial measures as assay indicators. Such phenotypic analyses have become extremely sophisticated, advancing screening enormously, but this approach can still be somewhat subjective. We describe the development, and validation, of a prototype high-content screening platform that combines steady-state fluorescence anisotropy imaging with fluorescence lifetime imaging (FLIM). This functional approach allows objective, quantitative screening of small molecule libraries in protein-protein interaction assays. We discuss the development of the instrumentation, the process by which information on fluorescence resonance energy transfer (FRET) can be extracted from wide-field, acceptor fluorescence anisotropy imaging and cross-checking of this modality using lifetime imaging by time-correlated single-photon counting. Imaging of cells expressing protein constructs where eGFP and mRFP1 are linked with amino-acid chains of various lengths (7, 19 and 32 amino acids) shows the two methodologies to be highly correlated. We validate our approach using a small-scale inhibitor screen of a Cdc42 FRET biosensor probe expressed in epidermoid cancer cells (A431) in a 96 microwell-plate format. We also show that acceptor fluorescence anisotropy can be used to measure variations in hetero-FRET in protein-protein interactions. We demonstrate this using a screen of inhibitors of internalization of the transmembrane receptor, CXCR4. These assays enable us to demonstrate all the capabilities of the instrument, image processing and analytical techniques that have been developed. Direct correlation between acceptor anisotropy and donor FLIM is observed for FRET assays, providing an opportunity to rapidly screen proteins, interacting on the nano-meter scale, using wide-field imaging.

## Introduction

High-content imaging can be defined broadly as the use of automated fluorescence microscopy with minimal user intervention, for screening of small-molecule libraries and interfering RNAs [1]. This is as opposed to high-throughput screening which concentrates on screening against huge libraries of compounds at high-speed; examples are flow cytometry [2], [3] or *in-vitro* assays using microplate-reader devices [4]. In recent years, specific instrumentation and software packages have been developed commercially for high-content screening. There are confocal, point scanning systems such as Opera (Perkin Elmer), ImageXpress ULTRA (Molecular Devices, Union City, USA) and the IsoCyte (Blueshift Biotechnologies, Sunnydale, CA, USA) and wide-field systems such as the ScañR (Olympus Soft Imaging Solutions, Germany). These are, for the most part, essentially microscopes which have been re-engineered to achieve a small footprint and full automation. Such systems allow phenotyping assays to be scaled-up to large numbers putting a huge burden on image analysis [5], [6]. Providing quantitative phenotypic information is extremely difficult [7], [8] and can be subjective.

The adoption of high-content screening by imaging puts the assay in a more physiological context by giving spatial information: a cell based assay can directly reflect the complexity of the myriad signalling pathways [9]. Many of these signalling cascades have been extensively investigated and the relationships between proteins have been partially delineated using biochemical techniques. Microscopical techniques, coupled with immuno-cytochemical methods allow us to preserve and image the relative localisation of multiple signalling molecules in cellular compartments under quiescent or stimulated conditions. Whilst a degree of localisation of proteins is conferred using these techniques, the spatial resolution afforded by conventional, far-field microscopy is insufficient to resolve the specific inter-relationship between individual protein complexes occurring on the nanometer length scale. Measurement of the near-field localisation of protein complexes may be achieved by the detection of Förster resonant energy transfer (FRET) between protein-conjugated fluorophores [10]. FRET is a non-radiative, dipole-dipole coupling process whereby energy from an excited donor fluorophore is transferred to an acceptor fluorophore in close proximity [11], [12], [13]. Clearly it would be desirable to combine the throughput of high content imaging with a quantitative read-out such as that generated in FRET assays.

In this paper we propose the use of two functional imaging modalities to provide quantitative information for high-content screening of protein-protein interactions using FRET. We use fluorescence acceptor anisotropy [14], [15] as a fast, first-pass screen, and combine this with fluorescence lifetime imaging [16], [17], [18] as a cross-checking methodology. The comparative strengths of the two techniques are discussed and the complementary nature of the metrics is elucidated. The first model system we test is the ‘Ras and interacting protein chimeric unit’ (Raichu), a Rho-GTPase FRET biosensor system kindly provided by Matsuda and co-workers [19], [20], and modified to express an eGFP [21] and an mRFP1 [22] acceptor making them suitable for imaging by fluorescence lifetime microscopy (FLIM) [23], [24]. Having previously applied acceptor anisotropy for dynamic imaging of such a biosensor in T-cells [25] we extend this assay to a high content screening format and use the Cdc42 version of the Raichu biosensor to demonstrate how this could be achieved. The 1∶1 stoichiometry makes Raichu particularly useful in this context since the analysis of FRET is made simpler by removing the effects of the relative concentration of the donor and acceptor molecules which occurs in dual expression systems [26]. We assay performance against a set of FRET standards consisting of eGFP and mRFP1 separated by amino-acid sequences of varying length (similar to those developed by Vogel and co-workers [27]). We use the test constructs to show that FRET imaged using acceptor fluorescence anisotropy and FLIM are highly correlated. This allows us to calibrate images generated using fluorescence anisotropy.

In a second model system we demonstrate how the technique can be applied to protein dimerization relying on the expression of two variants of the same protein with different fluorescent tags. In particular, we use cell lines stably expressing the chemokine receptor CXCR4, which is tagged to either monomeric eGFP or monomeric TagRFP [28]. CXCR4 receptor dimerization increases upon stimulation of the cells with the native ligand CXCL12 (formerly also known as SDF-1alpha) [29]. In a mock high content screen we exposed these cells to variety of small molecule compounds known to be effective inhibitors of several different cellular pathways. Using high-content FRET imaging, we were able to correctly categorize all small molecule compounds to either be CXCR4 inhibitors or not.

This paper represents the first demonstration of FRET efficiency measured by acceptor anisotropy and directly correlated with donor FLIM measurement of the same protein-protein interaction. The techniques presented here support the notion that not only trivial on/off FRET assays (such as DEVD substrate cleavage for caspase activity sensors) but also complex protein-protein interaction assays can be reliably sensed by acceptor FRET anisotropy. In combination these two techniques allows us to demonstrate that fluorescence acceptor anisotropy can be used as a fast, first-pass to screen FRET interactions which can then be cross-checked and accurately quantified using FLIM and to robustly identify false positives.

### Functional Imaging

#### FRET determination by acceptor anisotropy

A full discussion of the relationship between FRET and fluorescence anisotropy was described in detail elsewhere [14], [30], [31], [32]. Briefly, a measurement of the depolarisation of fluorescence of a molecular species can provide a sensitive measure of orientation, mobility and energy migration processes. In fluorescence anisotropy imaging contrast is obtained by monitoring the extent of polarisation retained by a population of molecules following excitation. It is an extremely versatile methodology and can be implemented in steady-state or time-resolved measurements and in wide-field or confocal microscopes. In a system of homogeneous molecules fluorescence anisotropy provides the only means by which self-interaction, (homo-FRET) can be measured [33]. In a hetero-FRET system it has been shown by us, and others, that fluorescence anisotropy can provide a high dynamic range method for monitoring energy transfer [15]. Fluorescence lifetime measurements of donor molecules undergoing FRET reveal a reduction in the radiative decay time since energy transfer provides an additional mechanism by which the molecules can return to their ground state. A reduction in fluorescence lifetime directly results in an increase in the polarisation, and therefore anisotropy, of the donor molecules in a hetero-FRET system as shown in the Perrin equation (1):
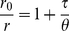
(1)Here r_0_ is the limiting (maximum) anisotropy, r the measured anisotropy, τ the fluorescence lifetime and θ, the rotational correlation time. However, the dynamic range of donor polarisation as a result of FRET is limited. Conversely, a low value of anisotropy of acceptor molecules is observed. The highly-polarised nature of the donor and the unconstrained orientation of the acceptor molecules results in an apparently highly depolarised acceptor population. This technique provides good dynamic range in a system where the fundamental anisotropy (i.e. in the absence of FRET) is high. Fluorescent proteins prove to be particularly good candidates since they have rotational correlation times that far exceed their fluorescence lifetime as they are large, sterically hindered molecules. Since the donors do not rotationally diffuse significantly before emitting a photon this results in maintenance of the initial photoselection of the dipoles to the polarised excitation.

Quantification of the acceptor anisotropy is analogous to sensitized emission FRET and was proposed by Jares-Erijman and Jovin in [34]:
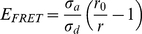
(2)Here *σ_a_* (*σ_d_*) is the acceptor (donor) extinction coefficient at the wavelength of excitation; *r_0_* is the fundamental anisotropy (in the absence of FRET) and *r* is the measured anisotropy.

#### Fluorescence lifetime imaging: donor FRET

In FLIM imaging contrast is generated by spatial variations in the excited singlet state lifetime of the observed fluorescent species. The sensitivity of this approach is such that FLIM microscopy has found wide-spread use in fields as diverse as pH sensing [35], viscosity and refractive index sensing [36], [37] and glucose monitoring [38]. FLIM also provides a means to measure FRET since the excited state population of the donor becomes depleted reducing both the fluorescence intensity and fluorescence lifetime of the donor. The advantage of using donor fluorescence lifetime to detect FRET is that the method is independent of fluorophore concentration, donor-acceptor stoichiometry and optical path length and is therefore well suited to studies in intact cells [39], [40], [41], [42], [43]. Combined with confocal or multiphoton techniques to examine the localisation of effects in cellular compartments, FRET/FLIM allows us to determine populations of interacting protein species on a point-by-point basis at each resolved voxel in the cell [44], [45], [46], [47], [48], [49]. The use of ‘ensemble’ FRET/FLIM techniques to probe protein-protein interactions in intact cells is now an established technique [50], [51], [52]. We and others have adapted the FLIM-based protein-protein interaction assays to directly monitor post-translational modifications (PTMs) ofproteins (such as phosphorylation by PKC [53], [54], [55], ubiquitination [56] and sumoylation [57], [58]) in live and fixed cells.

The measurement of fluorescence lifetime can directly inform on molecular proximity through the relation given in equation (3).
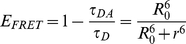
(3)Here *τ_D_* (*τ_DA_*) is the fluorescence lifetime of the donor in the absence (presence) of the acceptor. *R_0_* is the distance at which the FRET efficiency is 50% (the Förster radius) and *r* represents the intermolecular distance.

FLIM imaging of biological samples is currently undertaken using two distinct methodologies; a sequential single point measurement typically using the time-correlated single-photon counting technique (TCSPC) (but also frequency domain [59]) or a massively parallel detection using wide-field illumination and a gated or modulated intensified camera. The former approach, whilst offering excellent signal-to-noise and accurate lifetime determination suffers from a lack of speed due to both a requirement to scan and to integrate a sufficient number of photons to achieve an accurate fit to the data. In contrast, wide-field FLIM as exemplified by French and co-workers [60] or Bastiaens [61] (time and frequency domain respectively) is fast but suffers from both poor spatial resolution (due to the quality of available image intensifiers) and may be subject to artefactual errors in lifetime determination. In order to increase speed in laser scanning systems whilst maintaining a sufficient signal-to-noise ratio for FLIM applications, a number of multi-beam methodologies have been developed which enable parallelisation of image acquisition up to the maximum count rate of the detection electronics [62].

## Results

### Robustness and Sensitivity Testing

**Figure 1:**
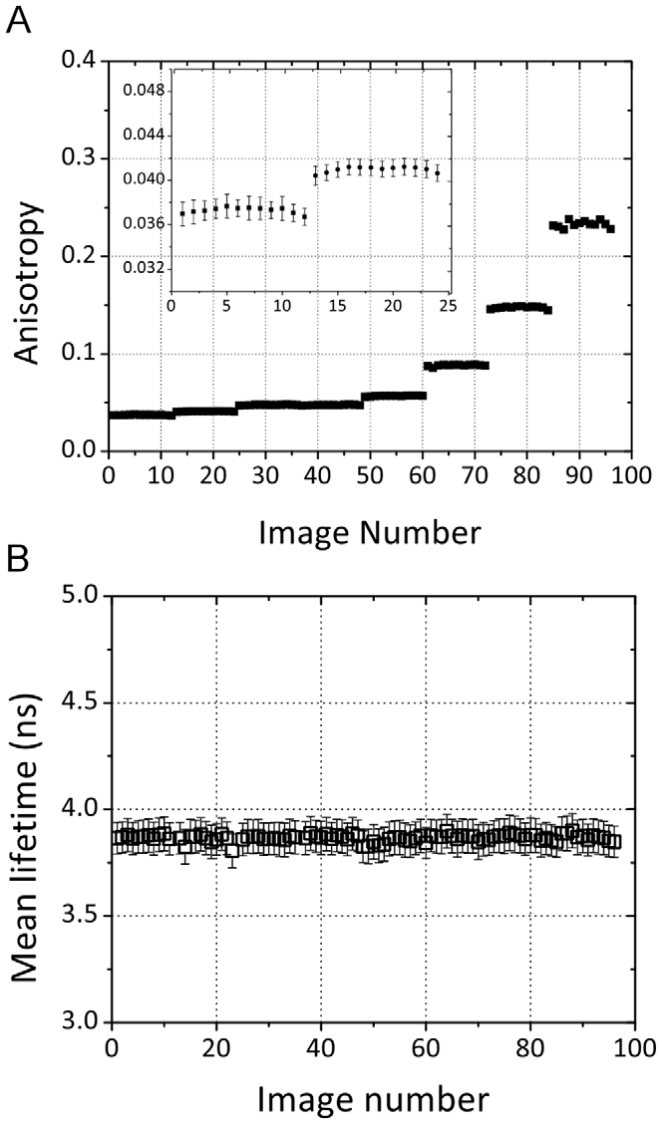
Demonstration of sensitivity and repeatability of fluorescence anisotropy and fluorescence lifetime imaging. (A) Anisotropy measurements of rhodamine B dissolved in varying concentrations of water and glycerol. The concentration of glycerol is used as a way of tuning the rotational diffusion, and therefore anisotropy, of the fluorophore. Any increasing percentage of glycerol reduces the mobility of the fluorophore molecules thereby increasing the fluorescence anisotropy. Differences of the order of 0.004 are easily and repeatedly measured. (B) Fluorescence lifetime measurements of the same rhodamine B sample. The concentration of glycerol has no effect on the lifetime. It is clear that the measurement is highly repeatable.

The sensitivity and robustness of the fluorescence anisotropy detection was characterised using a 96-well microlate containing Rhodamine B dissolved in solutions with varying concentrations of glycerol. Figure 1A, shows the full data range (a portion of data is given in the inset) from an experimental measurement of the fluorescence anisotropy. Since Rhodamine B is a small molecule, changing the concentration of glycerol allows the mobility, and therefore the anisotropy to be continuously varied. These data illustrate the sensitivity and repeatability achieved. Each image in the sequence is acquired from individual wells the microplate with the concentration of glycerol being varied in each of the eight rows of the plate. The sensitivity is such that a change in anisotropy of ±0.004 is clearly and repeatedly detected.

The FLIM component of the microscope was tested by measuring the fluorescence lifetime in each well of the same Rhodamine B sample (as shown in figure 1B). Although the fluorescence anisotropy is dramatically altered by varying the glycerol content, the fluorescence lifetime remains constant. The graph shows that there is little variation in the decay time across the plate when fitted to a mono-exponential function.

### FRET Standards

**Figure 2:**
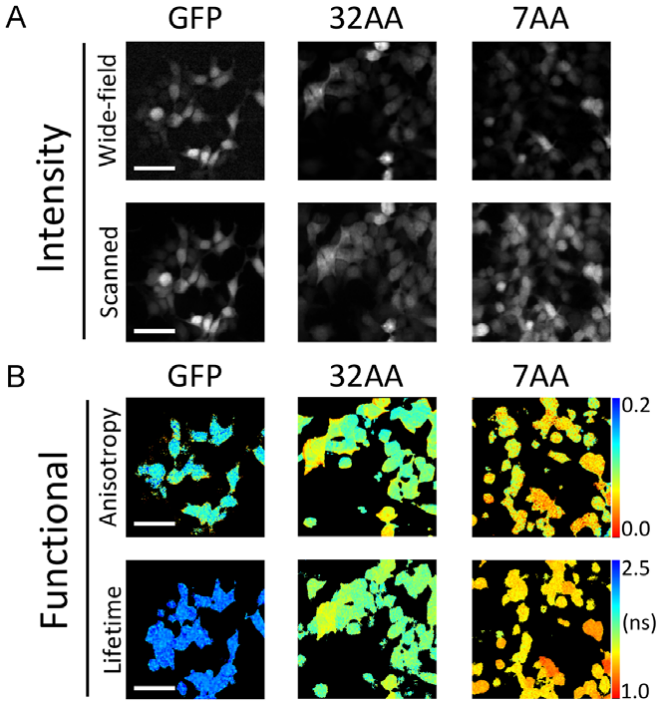
Imaging of FRET standard constructs expressed in 293T cells. (A) Intensity images from the wide-field and laser scanning modalities. (B) Functional images for GFP, 32AA and 7AA standards. This amply demonstrates the correlation between the two measurement techniques and the sensitivity in determining changes in FRET efficiency. (Scale bars represent 50 µm.)

FRET standards, similar to those described by Vogel and co-workers [27], consisting of eGFP linked to mRFP1 by amino acid chains of varying lengths (7, 19, and 32 amino acids) are used both as a means to correlate the acceptor anisotropy and donor lifetime measurements, and to provide positive and negative controls in screening data. Figure 2 shows example images of 293T cells transiently transfected with the 7AA and 32AA constructs and plated onto coverglass (see Materials and Methods). Figure 2A shows intensity images from the wide-field and laser scanning components of the microscope. Figure 2B illustrates the correlation between the anisotropy and lifetime imaging techniques. Although there is some cell to cell heterogeneity in the images, due to a variation in transfection efficiency across the sample, it is clear that the anisotropy and lifetime images provide identical contrast.

**Figure 3:**
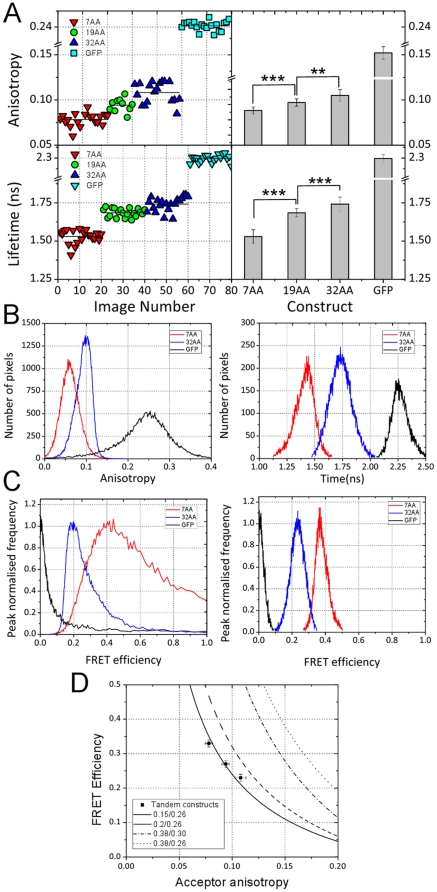
Summary of FRET standard data. (A) Examples of histograms produced from functional images. Mean values from these images are compared across the data set in screening experiments. (B) Plot of equation (2) showing a proposed means for converting sensitised emission anisotropy to FRET efficiency. (C) Histograms from (A) after conversion to FRET efficiency. (D) Accumulated data from multiple regions of interest from FRET standard imaging. Mean values from the functional images are plotted for each construct illustrating the scatter in the data. The average value obtained for each construct is shown as a column plot.

The data for all three FRET standards in both modalities are summarized in figure 3A. In the FLIM data an increasing FRET efficiency is observed with a decrease in linker length with values of 23%, 27% and 33% measured for the 32AA, 19AA and 7AA constructs respectively. A corresponding, and correlated trend (see figure S1) is seen in the anisotropy data. Despite the fact that the photophysics of the hetero-FRET interaction limits the dynamic range of the measurement, the sensitivity of the technique is such that variations in anisotropy, which directly result from a difference in the rate of energy transfer, can be observed with identical contrast to that observed in the FLIM data.

Histograms from the representative anisotropy and lifetime images in Figure 2B are shown figure 3B. Despite the heterogeneity present in the images a clear distinction can be seen in the histograms. The GFP histogram for the anisotropy data is somewhat wider than those for the 7AA and 32AA constructs since these data were calculated from GFP bleed-through in the RFP channel. The low number of collected photons results in a broad histogram. These data were converted to FRET efficiency using equation (2) for the anisotropy and using equation (3) for the lifetime data (figure 3C).

### High-Content Screening

#### Small molecule inhibitor screening: CDC42 Riachu Biosensor

**Figure 4:**
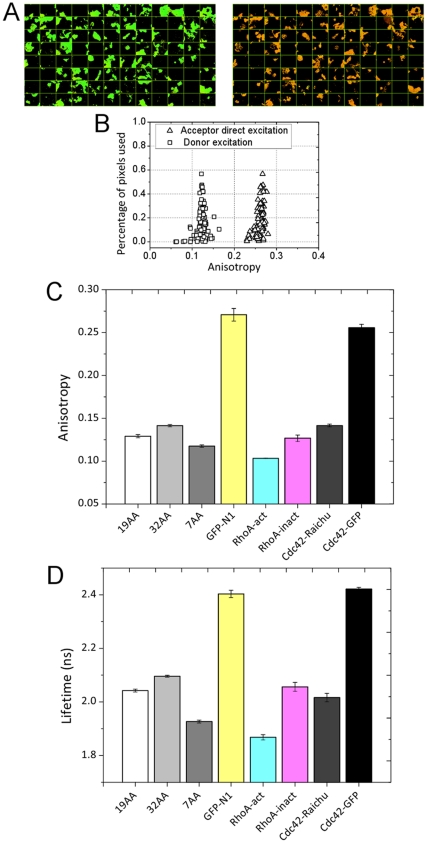
A comparison FRET standard constructs to the baseline activity of Raichu and RhoA biosensors. (A) Anisotropy measurements of Cdc42-Raichu biosensor stably transfected in A431 cells when the RFP acceptor is both directly and indirectly excited. The baseline activity of the biosensor results in apparently depolarised sensitised emission with an average value of 0.125±0.009 as compared to 0.263±0.006 when directly excited. (B) Plot of anisotropy values versus the percentage of the image pixels used in the calculation. This provides an extra metric by which outliers can be removed from the data. (C) Fluorescence anisotropy imaging data. (D) Fluorescence lifetime imaging data. The error bars in both graphs represent the pooled standard deviation of 3 images (in each modality) for each condition.

Figure 4A shows examples of acceptor fluorescence anisotropy of the Cdc42-Raichu biosensor probe when stably expressed in the A431 human epidermoid cancer cell line and seeded in a 96 well microplate. Upon binding of GTP to Cdc42, the biosensor changes conformation bringing the donor eGFP in close proximity to the acceptor mRFP1 resulting in a reduction of the donor fluorescence lifetime and an apparent depolarisation of the acceptor emission. One image per well was acquired when exciting the acceptor directly and then indirectly via the eGFP donor. This provides a means to characterise the level of heterogeneity in the baseline measurement of acceptor anisotropy and to test the ability of the microscope to measure sensitised emission. We can also test the level of bleed-through of donor emission into the acceptor channel, and vice versa. The data points in the graph plotted in figure 4B summarise the acceptor anisotropy when directly excited and when excited through energy transfer which plots fluorescence anisotropy as function of the image area used in the calculation. The instrument operates in an automated, unsupervised mode where the coordinates for image acquisition are generated by capturing the positions of seven separate points across the plate; the software produces a grid of (*x, y*) coordinates and a polynomial function is used to generate the *z*-position (or focal plane). This is necessary due to the non-linear topography of the cover-glass of the multi-well plate. These (*x, y, z*) coordinates are visited in turn and a software autofocus routine is used to provide a fine adjustment of the focal plane prior to acquiring an image. Currently, we use the image area above a given intensity threshold as a percentage of the total imaged area as a means to exclude empty images from subsequent analysis. After removal of such outliers, we measure a mean value across the 96-well plate of (0.263±0.006) and (0.125±0.009) when excited directly and via the donor (by energy transfer) respectively (figure 4B).

Figure 4C and 4D shows the fluorescence anisotropy and lifetime of a selection of biosensor constructs as well as the three FRET standards. All constructs were expressed in 293T cells which were then plated in 24 wells of a 96-well microplate and fixed as described in Materials and Methods. This experiment illustrates the process of screening using functional imaging. Here we measure both the anisotropy and the fluorescence lifetime for four regions of interest for each of the 24 wells giving eight different conditions with three repeats of each condition. All the information required to calculate the acceptor anisotropy is captured in 800 ms CCD exposures and averaging over three frames. With an autofocus time of 6 seconds and the time taken to move the microscope stage between the coordinates the epi-fluorescence images from which the anisotropy is calculated are acquired within 17 minutes. The fluorescence lifetime images by comparison require three minutes to capture a single frame and therefore acquiring all 108 images takes 5.4 hours.

**Figure 5:**
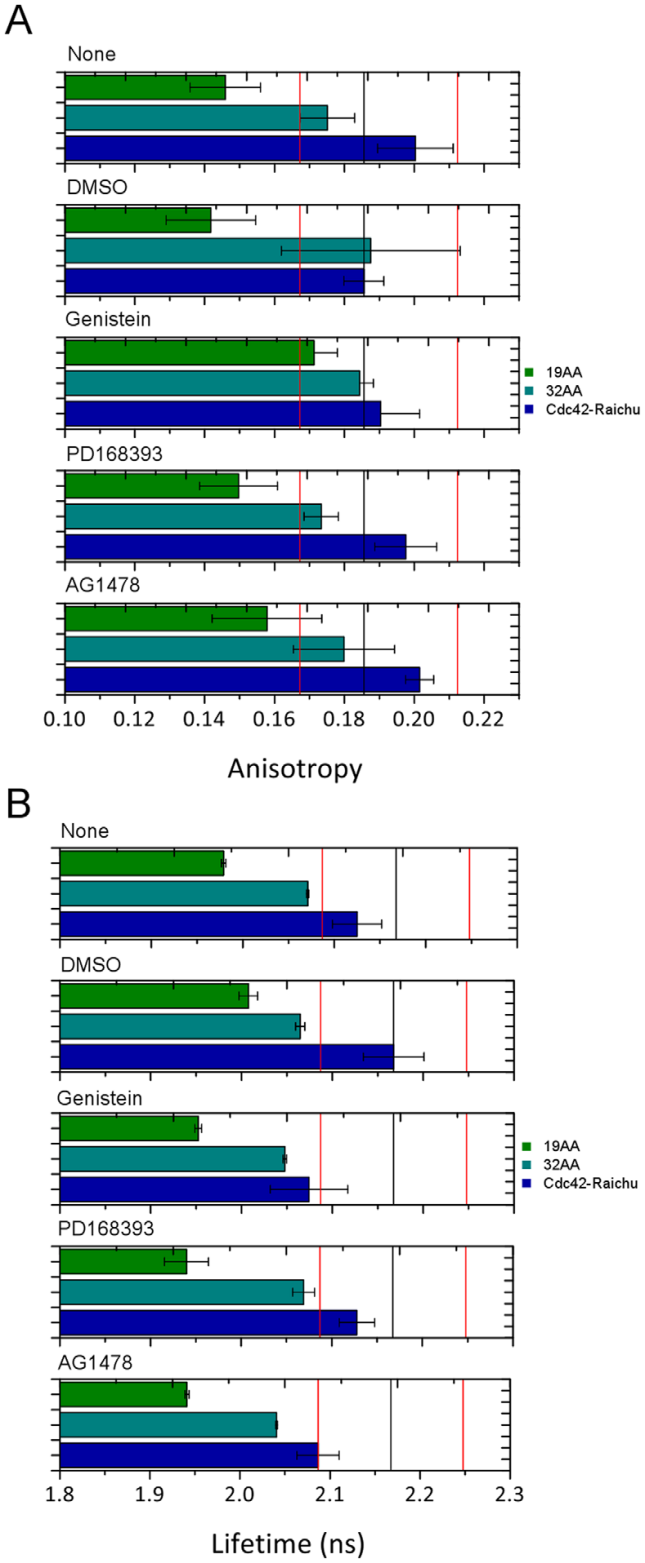
Direct comparison between (A) acceptor FA and (B) donor FLIM when screening TKIs against the Cdc42-Riachu Biosensor. The error bars in both graphs represent the pooled standard deviation of 5 images (in each modality) for each condition.

We tested our methodology by studying effect of tyrosine kinase inhibitors (TKI) on Cdc42 using the Raichu biosensor system. Figure 5 shows data from 50 wells of 96 well microplate seeded with A431 epidermoid cancer cells. In three wells per row (total of 15 wells), the cells were transiently transfected with FRET standard constructs (data for 19AA and 32AA shown) and four wells per row (total of 20 wells) contained Cdc42-Raichu. Three rows of the plate were treated with a different TKI, one row remained untreated and one row was control treated with DMSO (the solvent in which the TKIs are dissolved). Five images per well in each modality were recorded. The wide-field images were recorded using 800 ms exposures and three frames were averaged to provide adequate signal-to-noise for calculation of senstised emission anisotropy. All the information required to calculate the fluorescence anisotropy was obtained in around 43 minutes (including the time taken for the autofocus routine to run). The FLIM data required 3 minutes per image and a total time of 12.5 hours for the entire data set.

#### Small molecule inhibitor screening: Chemokine receptor dimerisation

The chemokine receptor CXCR4 is a member of the transmembrane domain G protein-coupled receptor superfamily (GPCRs) which is specific for the CXCL12 ligand. CXCR4 has been shown to act as a co-receptor for HIV [63] and is highly expressed in human breast cancer cells [64]. CXCR4 is an excellent model system since it forms homo-dimers during CXCL12 stimulated internalisation, a process which can be visualised using FRET [65], [66]. Prior to stimulation CXCR4 is located at the cell membrane and does not undergo dimerisation. We used a rat breast cancer cell line (MTLn3E) stably co-expressing CXCR4-eGFP and CXCR4-TagRFP, generated by retroviral infection and subsequent single cell sorting by FACS to obtain clonal populations with defined ratios of the two differently tagged receptor molecules. Upon ligand treatment a mixed population of homo- (CXCR4-eGFP only and CXCR4-TagRFP only) and hetero-dimers (CXCR4-eGFP and CXCR4-TagRFP) are to be expected but here we select the hetero-FRET sub-population by means of our chosen measurement methods. In order to increase the probability of measuring this sub-population we used a clone with an acceptor (CXCR4-TagRFP) to donor (CXCR4-eGFP) ratio of 4∶1 for all experiments.

**Figure 6:**
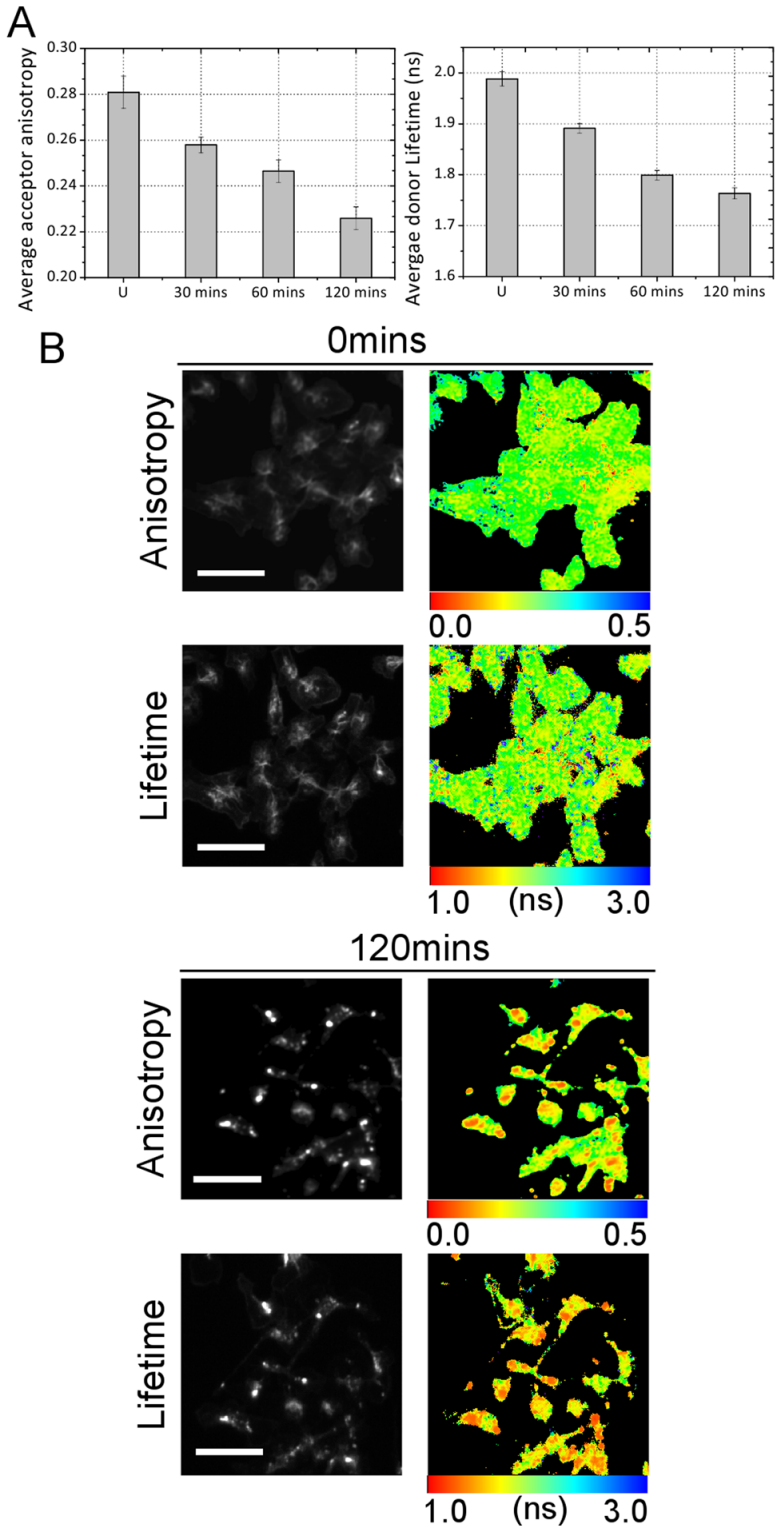
Time course data showing the change in FRET efficiency that occurs upon the internalisation of CXCR4 after treatment with CXCL12. (A) Column plots for acceptor anisotropy and donor FLIM for each time point (average of ten images per time point). The error bars represent the pooled standard deviation from multiple images (B) Example functional images showing change in FRET efficiency upon interalization and correlation between the two imaging techniques. (Scale bars represent 50 µm.)

In figure 6 we demonstrate that CXCR4 dimerisation can be monitored using both fluorescence anisotropy and FLIM imaging in cells fixed at various time-points after treatment with CXCL12. The plots represent the pooled image mean for ten images per condition. At time zero the values of acceptor anisotropy (0.28 for sensitised Tag-RFP) and donor lifetime values (2.0 ns) attest to the fact that there are a low number of dimers prior to internalisation. After 120 mins these values drop to 0.23 and 1.75 ns indicating an increase in FRET efficiency upon treatment with the ligand.

**Figure 7:**
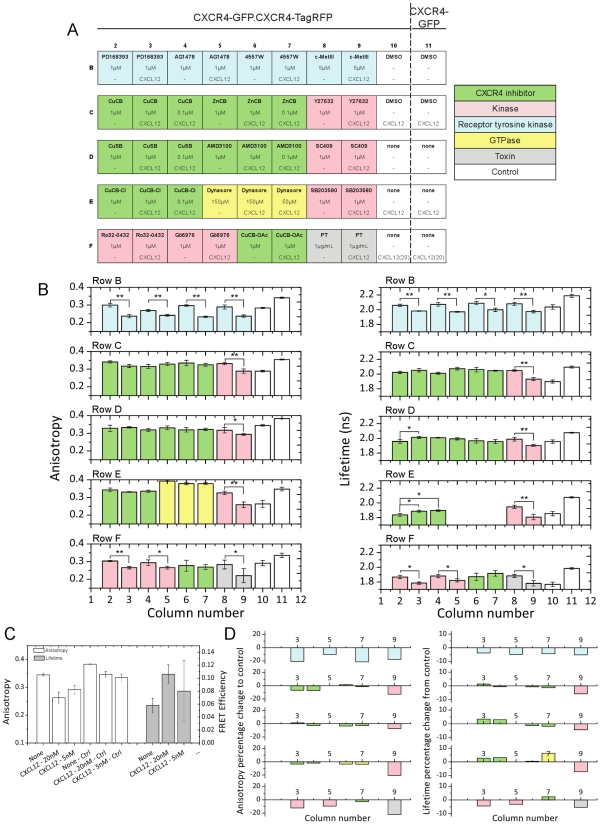
Summary of small molecule inhibitor screening data of CXCR4. (A) Layout of microplate. Wells marked in green are CXCR4 inhibitors. Controls are shown in white: column 10 shows data for CXCR4-eGFP and CXCR4-TagRFP transfected cells without inhibitor treatment; column 11 shows CXCR4-eGFP only cells without inhibitor treatment. In column 10 the concentration of CXCL12 is varied: row D is untreated while rows E and F are treated with 5 nM and 20 nM CXCL12 respectively. (B) Comparison of anisotropy and lifetime data showing that dimerisation and internalisation is blocked by CXCR4 inhibitors. Errors bars represent the standard deviation of repeated measurements in each well (4 images per well for each modality). (C) Plots of anisotropy and FRET efficiency for column 10, rows D, E and F: cells in the absence of any inhibitors (CXCL12 concentrations of 5 nM and 20 nM). (D) Percentage change in anisotropy and lifetime compared to controls.

Subsequently, we performed a small molecule screen with the aim of identifying compounds blocking CXCR4 dimerisation. In this mock experiment we used CXCR4 inhibitors alongside kinase, receptor tyrosine kinase and GTPase inhibitors (figure 7A). 50 wells of the 96 well microplate were seeded

with cells, each row of the plate being internally controlled. The assay is performed in the presence or absence of the CXCR4 ligand CXCL12 in order to obtain a control measurement for each inhibitor. The anisotropy and fluorescence lifetime data are summarised in figure 7B which shows the pooled data for 5 images in each modality for each condition. The colours of the data bars indicate the treatment condition of each well (as shown in figure 7A).

To summarise the data, firstly, figure 7C shows that the acceptor anisotropy and FLIM data of cells in the absence of any inhibitors (varying CXCL12 concentrations) are in agreement showing the expected concentration-dependent increase in receptor dimerization (these data are displayed in column number 10 of the graphs of figure 7B). In this case we choose to convert the lifetime data to FRET efficiency since a row-to-row variation occurs in donor lifetime data. However, the inclusion of CXCR4-eGFP only control wells within the row (column number 11 in figure 7B) meant that a conversion to FRET efficiency could be used to internally control for this effect. In the absence of the acceptor construct CXCR4-TagRFP (i.e. CXCR4-eGFP only) (column number 11 of figure 7B), we do not measure any FRET by fluorescence acceptor anisotropy or FLIM as expected. Furthermore, we find that all dedicated CXCR4 inhibitors (green wells in figure 7A and 7B) are highly effective in blocking dimerization as both the fluorescence acceptor anisotropy and lifetime values do not drop below that of the untreated wells (unstimulated controls). Those compounds proved to be at least equally effective as the commercially available CXCR4 inhibitor AMD3100. These findings are summarised in figure 7D which plots the percentage change against the control wells for both the anisotropy and lifetime data. This graph clearly shows which compounds were effective in blocking CXCR4 internalisation. The wells where a change in FRET efficiency upon treatment with CXCL12 are clearly visible in these graphs. It is also clear that all wells where the dedicated CXCR4 inhibitors were applied did not show statistically significant changes in FRET, as compared to the wells untreated with CXCL12. These wells can be easily identified in either methodology. It should be noted that the numerical values of the percentage changes are not the same in each methodology. As discussed earlier, reductions in anisotropy and lifetime are highly correlated, however the relationship between anisotropy and FRET efficiency are highly non-linear and therefore we would not expect percentage changes in anisotropy and lifetime to be linear.

The dynamin inhibitor (dynasore) was used to test accumulation of receptor dimers at the plasma membrane in the absence of receptor internalization. Dynamin has been shown to be required for the endocytosis of a number of receptors [67], [68], including that of GPCRs [69]. However, our measurements suffered from a lack of correlation between the anisotropy and lifetime results due to the dynasore compound being highly fluorescent (see figure S2). The fluorescence which appears in the donor emission channel causes a significant reduction in the measured fluorescence lifetime for these wells (0.7 ns) but the measured anisotropy in the acceptor channel is around 0.4. Figure S1 shows that in fact the compound is distributed throughout the (glycerol based) mounting medium which has effectively immobilised the molecules leading to the high anisotropy value. Importantly, although producing spurious data in the functional imaging modalities, we could conclude that dynasore was ineffective in blocking receptor internalisation since vesicle formation upon ligand treatment could clearly be visualised in the wide-field intensity images (from which the anisotropy is calculated).

## Discussion

### FLIM and Anisotropy Measurements of FRET are Correlated

The FRET standard constructs provide us with positive and negative controls for the biosensor assays. In the case of the 7 amino acid linker the eGFP and mRFP1 a calculation of the Forster radius estimates the fluorophores to be within 5 nm: a distance which is unlikely to be achieved in the case of a biosensor molecule. The FRET efficiency of the 32AA construct appears similar to the base-line activity of the biosensors, where the construct would be in an inactive confirmation. It would appear, then that in its inactive conformation the biosensor is not what we could consider to be fully open. We can draw this conclusion since the biosensor is a molecule of 3422.82 Daltons with a full length which far exceeds the 32AA construct. To make a direct comparison to the Raichu biosensor would require a much longer linker length, which would be unfeasible due to the flexibility of the amino acid chain. Nevertheless, in a real assay these constructs can be used to provide additional positive and negative controls.

Figure 2 illustrates that when the spatial information is retained in the anisotropy the correlation with FLIM imaging data is remarkably good. A pixel-by-pixel analysis is difficult since the anisotropy data is obtained using a single CCD frame with the microscope in epi-fluorescence mode and the FLIM image is captured by laser scanning: the two modalities are not perfectly registered due to the sampling and image magnification. However, despite the anecdotal nature of the comparison it is clear that the two modalities are very well correlated. Figure S1 shows an analysis of the correlation on an image-by-image basis. Pearson’s coefficient is calculated to be 0.75 (when discounting the eGFP alone data) showing the two methodologies to be highly correlated. This gives us confidence that an acceptor anisotropy measurement will reproduce any variations in FRET efficiency that might arise in the screening assays allowing subsequent quantification using fluorescence lifetime measurements.

The estimation of FRET efficiency given in equation (2) requires knowledge of the molar extinction coefficients for both donor and acceptor molecules which, like the reference lifetime of the donor fluorophore, are difficult to determine in-situ and require calibration samples or a reliance on literature values. In figure 3D, equation (2) is plotted using literature values of the extinction coefficients and using absorption data obtained for mRFP1 in neutral buffer [45] at the excitation wavelength (490 nm) and measured values of donor anisotropy in the absence of acceptor. The data points plotted consist of FRET efficiency, determined using fluorescence lifetime measurements, plotted against acceptor anisotropy for the FRET standard constructs. It is clear that a very similar trend is achieved between equation 3 and the experimental data. However, fitting the data requires a significant alteration of the mRFP1 extinction coefficient from the spectroscopic data and literature values. It is interesting to note that the absorption spectrum of mRFP1 contains a peak corresponding to absorption of immature or poorly folded protein centred at approximately 500 nm. Given that this state does not appear in the excitation spectrum and may vary dependent on level of expression relative to the principle excitation peak, we might reasonably assume that the calculated value for the mRFP1 extinction coefficient incorrect_._ This is somewhat unsurprising since the equation suggested in [34] is really only applicable in systems which are dynamically averaged (the incorrect assumption being that the acceptor will fully depolarise within the lifetime of the donor excited state). Additionally, the equation is poorly behaved at the limits of low and high FRET efficiency and inevitably produces an artificially broad histogram when converting anisotropy imaging data to FRET efficiency (Figure 3B). It is clear that there are many unknown factors which prevent us unambiguously converting acceptor anisotropy to FRET efficiency and it is beyond the scope of this work to produce a theoretical framework within which this could be done.

Figure 4 shows a comparison of the FRET standard constructs with several Rho-GTPase biosensor constructs, all with eGFP as the donor fluorophore and mRFP1 as the acceptor. Figure 4 A and B directly compares the output from an acceptor anisotropy measurement and a corresponding donor lifetime measurement for the constructs. These data show that the correlation between the two techniques is apparent for all the constructs tested. This gave us the confidence that such an approach could be used in a biosensor screen.

### Cdc42-Riachu Biosensor Pseudo Screen

Figure 5 shows a comparison of fluorescence anisotropy and lifetime data for a small-scale test screen of three TKIs against Cdc42-Raichu. A431 cells were plated in 60 wells of 96 well plate and five images per well were acquired in each modality. In this experiment the anisotropy and lifetime did not exhibit as high a correlation as observed in the previous data sets. This proved to be due to a sample preparation dependence in the lifetime data which was not reproduced in the acceptor anisotropy data. The inclusion of the 19AA and 32AA constructs prove that the reduction in lifetime seen in the Cdc42-Raichu data is not a result of addition of the TKIs. These data show a gradual reduction in lifetime (and so an increase in notional FRET efficiency) for all conditions including the FRET standards and DMSO control. Clearly, since the FRET standards are inactive they are not expected to be affected by addition of the TKIs. Therefore the inclusion of two modalities which we know to be highly correlated (as shown by the FRET standard data given in figures 2 and 3) allows us to effectively identify the presence of false positives and sample preparation dependent effects in any FRET screen.

Previous experiments using high-resolution multi-photon FLIM (data not shown) have suggested that the TKIs AG1478 and PD168393 should produce an increase in Cdc42-Raichu activity in this assay but this result was not recapitulated in this screen. We performed additional live-cell FLIM experiments (figure S3) to check the biosensor activity. Three clear phenotypes are seen in these experiments. Those cells that are highly motile have a high FRET efficiency indicating that the Cdc42-Raichu biosensor is highly active. Cells undergoing mitosis also show high activity of the probe. After division and as junctions are formed with other cells biosensor activity reduces to something like baseline levels. This provides compelling evidence for a lack of hits in the screen. The samples are prepared such the cells are grown to a confluent monolayer before fixation in order to provide good coverage of the well. Although there is a proportion of cells where the probe was highly active when fixation occurred, most of the cells in the image are quiescent and therefore the biosensor is at baseline levels of activity. It is clear that the experiment design needs modification in order to find hits in this kind of screen. However, this does not detract from the fact that the methodology of using a combination of acceptor anisotropy and donor is very effective.

### Acceptor Anisotropy Quantitatively Reports Protein-protein Interaction

Where 1∶1 donor:acceptor stoichiometry does not exist, as in the CXCR4 assay the situation is complicated by the presence of unbound donors and acceptors. This case where we have mixed populations of FRET pair (CXCR4 dimers) and free donor and acceptor molecules is similar to the p-FRET concept presented in [70]. Here we do not follow Mattheyses et al. and produce an estimate of the relative concentrations of the three populations since we are interested only in whether CXCR4 internalisation, and therefore dimerization, is inhibited. This is a fairly unambiguous assay since binding of CXCL12 to CXCR4 produces highly distinct vesicular structures. We seek to generate a readout of this process without the need to examine whether these structures are produced but of course the fact that we have the images available to us means we can always cross-check our result. In other assays where the FRET pair does not undergo such obvious translocation it would be necessary to pre-calibrate the assay such that the fluorescence anisotropy of the donor, acceptor and FRET-pair separately so that an accurate measure of the interaction is produced. The lifetime data in figure 6 show that the FRET efficiency is around 10% when dimers are formed upon internalization. It is important to note that the sensitivity of the anisotropy measurement is sufficient to give a robust readout even in this assay. We were also able to reproduce the same hits unambiguously using both wide-field and scanning methodologies. The dedicated CXCR4 inhibitors could be identified by either methodology and again each technique proved to be highly correlated. We were also able to reproduce a concentration dependence in the application of CXCL12 (Figure 6C).

In order to screen for potential inhibitors of CXCR4 it was necessary to engineer a donor and acceptor expressing cell line as a biosensor to determine the effect of a population of receptors which is reproducible and effective across the cell population for a variety of treatments. In this case, we do not measure a change in FRET efficiency in the purest sense (since the interaction distance between donor and acceptor is believed to be similar whether a dimer is formed on the membrane or within a cellular vesicle). Instead, we expect a change in the interacting population. In a cell with uncontrolled levels of donor and acceptor this interacting population may be determined in the case of fluorescence lifetime imaging by attempting to fit biexponential models to the data (thereby assuming only two populations: interacting and non-interacting for the donor). In this case the comparison between different cell populations (treated and untreated) becomes significantly more complex since both the stoichiometry and the interacting fraction must be known for each cell. Also the calculation of FRET efficiency from a single exponential fit is significantly more robust than a biexponential at low photon counts. If we control the stoichiometry whilst maximising dynamic range (increasing the probability of green-red dimer formation) and maintaining function of the protein of interest, we have a robust cell biosensor. In this case single cell clones containing CXCR4- eGFP and CXCR4-TagRFP were screened for both functionality and FRET efficiency with/without stimulation to determine the best compromise between expression level and FRET dynamic range (a 4∶1 ratio was found to be near optimum). In adapting this assay for other protein-protein interactions the optimum stoichiometry must be determined through cloning, cell sorting and screening operations for function and dynamic range. It is clear that fluorescence anisotropy provides a fast metric which can be used for hit finding in high-content screening assays. It should be noted that although the FLIM images require significantly longer acquisition times they still not prohibitively long. Should lifetime data be required for the entire microwell plate, as in the assays presented here, the experiment can simply be performed overnight or through the course of several days since the microscope platform can run completely unsupervised. With advances in detection technology and more advanced data fitting routines these acquisition times will improve dramatically. New hybrid detectors available from Hamamatsu provide significant improvements in sensitivity and should reduce acquisition times (for the same number of collected photons) by at least 50 percent. The application of Bayesian or maximum likelihood statistics to data fitting will reduce the number of photons required to determine the fluorescence lifetime. Preliminary studies suggest that acquisition times could be reduced to less than one minute per image making lifetime imaging a viable screening methodology in its own right. Here we have applied the two techniques in FRET assays using a low NA microscope objective lens so that we could collect data from a statistically significant number of cells. The techniques could equally apply in a situation where high-spatial resolution, and therefore high NA lenses would be required (such as measurement of FRET within microdomains). However, there is a caveat to be considered in the case of fluorescence anisotropy: high NA lenses result in a depolarisation of the fluorescence emission and, therefore, must be considered. A correction for depolarisation is easily calculated and has been published previously by Axelrod et al [71].

## Materials and Methods

### Plasmids, Cell Lines, and Cell Culture Conditions

mRFP-eGFP FRET standards were constructed by the ligation of mRFP1 into the pEGFP-N1 vector used in the manufacture of the Raichu biosensor constructs, generating random amino acid linkers. The linker for the 7 AA sequence is RDPPVAT, for the 19AA sequence is KLRILQSTVPRARDPPVAT, and that for the 32AA sequence is KLRILDITSGLETRDASGQSTVPRARDPPVAT. These constructs were expressed transiently in human embryonic kidney (HEK)-293T cells [72] obtained from the American Type Culture Collection (ATCC).

Raichu probes for reporting the spatial activity of small Rho GTPases, obtained from Prof M. Matsuda (Osaka University, Japan), were modified from their original form to replace the YFP/CFP fluorophores with eGFP/mRFP1 such that the probe can be used in fluorescence lifetime imaging [24]. The resultant GFP- and mRFP1-tagged Raichu constructs retain the original design of the CFP/YFP-tagged version i.e. mRFP1-PBD-GTPase-GFP12. The sensor was synthesized by excision of the module between YFP and CFP and insertion between the mRFP1 and eGFP (in the pEGFP-N1 vector (Clonetech), modified by the addition of mRFP1 and altering the multiple cloning site). The membrane targeting CAAX sequence was added after the eGFP. A431 human epidermoid cancer cell lines [73] (obtained from the ATCC) were generated stably expressing the Cdc42 variant of the biosensor and Cdc42-eGFP to provide a donor-only control.

The chemokine receptor CXCR4 was fused with its C-terminus via a short linker to the N-terminus of either GFP or TagRFP as described earlier [66], [74]. Briefly, constructs were obtained by sub-cloning the human CXCR4 coding sequence into the HindIII and EcoRI sites of pEGFP-N1 (Clontech) or pTagRFP-N1 (Evrogen), followed by further sub-cloning into the retroviral expression vectors pLPCX or pLHCX (both from Clontech), and verification by sequencing. MTLn3E mammary rat carcinoma cells [75] (a kind gift of Dr Erik Sahai (London Research Institute, Lincoln’s Inn Fields)) stably expressing human CXCR4-eGFP were obtained after retroviral infection, selection with puromycine (2 µg/mL), and fluorescence-activated cell sorting (FACS) in order to obtain single clones. A cell line expressing both fluorescent proteins was generated via sequential retroviral infection with pLPCX CXCR4-eGFP and pLHCX CXCR4-TagRFP, selection and FACS-based single clone sorting. One single clone of each cell line was selected and used for all further experiments.

All clones were cultured in αMEM supplemented with 5% fetal bovine serum, penicillin/streptomycin (100 IU/0.1 mg/mL), and *L*-glutamine in an atmosphere containing 5% CO_2_/95% air (v/v) in the presence of the respective selection antibiotics (0.5 µg/mL puromycin and/or 200 µg/mL hygromycin).

### Reagents

Recombinant CXCL12 was obtained from Peprotech and generally used at 100nM (unless otherwise indicated). The following kinase inhibitors were purchased from Calbiochem (targets in brackets): Ro32-0432 and Gö6979 (both protein kinase C); SB203580 and SC409 (p38 MAP kinase); SL327 (MEK1 and MEK2 kinases); PP2 (SRC kinase); wortmannin (phosphatidylinositol-3-kinase); Y27632 (Rho associated protein kinases); AktVIII (AKT1 and AKT2 kinases); dynasore (GTPase activity of dynamin1/2); AG1478, PD168393 and Genistein (EGFR receptor tyrosine kinase); 4557W (EGFR and ErbB2 receptor tyrosine kinases); c-MetIII (c-MET receptor tyrosine kinase). The bicyclam compounds CuCB, CuCB-OAc, CuCB-Cl, ZnCB, and CuSB were kind gifts of SJ Archibald and are intended to inhibit CXCR4 [76], [77]. The CXCR4 inhibitor AMD3100 and Pertussis Toxin (PT) were from Sigma.

### Sample Preparation for Imaging

For anisotropy and/or FLIM experiments cells were plated either onto glass cover slips (No.1.5, Mentzel) or into black glass-bottom 96-well microplates (Greiner). All glass surfaces were acid-washed (40% (v/v) conentrated HCl in ethanol) and sterilized before cell seeding. If desired, sub-confluent cells were pre-treated with inhibitors or toxins at the indicated concentrations for 60 min. Cells were treated with CXCL12 as indicated (100 nM, unless stated otherwise), fixed with 4% (w/v) paraformaldehyde at room temperature for 15 min, permeabilized with 0.25% (v/v) Triton X-100 in PBS for 10 min, treated with 1 mg/mL NaBH_4_ in PBS for 5 min, rinsed with PBS, and mounted in MOWIOL containing 2.5% (w/w) DABCO as antifading agent.

### Automated Microscopy

The combined fluorescence lifetime and fluorescence anisotropy microscopy system has been described elsewhere (recent publications by Matthews et. al [25], Barber et al [78]) and online (http://users.ox.ac.uk/~atdgroup) but in brief:

The microscope has two, user selectable, modes of operation. An epi-fluorescence microscope is constructed using off-the-shelf optical elements into which is coupled the output from a halide lamp using a liquid light guide (Intensilight, Nikon Instruments, Japan). To achieve anisotropy imaging the output from the lamp is passed through a linear polariser and half-wave plate. Fluorescence is detected via a QuadView polarisation resolved image splitter (Photometrics) which collects light in two orthogonal polarisation channels at wavelengths of 515±30 nm and 630±70 nm (filters obtained from Chroma Technologies, Rockingham, VT, U.S.A.); wavelengths which have been selected for the detection of eGFP and mRFP1. In a single CCD exposure we obtain four spatially identical images, which differ according to their wavelength and polarisation content. The four sub-images are extracted and used to calculate fluorescence anisotropy of the donor and acceptor fluorophores. To enable automated operation we include a motorised stage (Märzhauser GmbH, Wetzler, Germany), a closed-loop objective lens mount with a 500µm range of travel (Piezosystem Jena GmbH, Jena, Germany), and a motorized filter cube selector. The system is controlled using an integrated modular software package developed providing the user with the ability to control every aspect of image acquisition including access to the Python scripting language for complete automation of an assay or screen. Currently, post-acquisition image processing is performed off-line using ImageJ and MATLAB.

An afocal dual axis scanning system and time-correlated single photon counting (TCSPC) detection (SPC830, Becker and Hickl) is incorporated to enable fluorescence lifetime imaging. Fluorescence excitation is provided by a 473 nm emitting laser diode (BDL-473_SMC, Becker and Hickl) which generates optical pulses with a duration of 40 ps at a repetition rate of 80 MHz. Single photon, time-resolved detection is achieved using a photomultiplier tube (PMH-100, Becker and Hickl) filtering for the fluorescence with a 510±30 nm bandpass filter for eGFP. To avoid pulse pile up and to ensure photobleaching is kept to a minimum count rates are maintained at less than 1×10^5^ events per second.

FLIM by TCSPC does not achieve the imaging speed of wide-field frequency domain systems but is the gold-standard for lifetime imaging due to its accuracy and time-resolution. It is for this reason, and given our previous experience with such methodologies, that we incorporate lifetime imaging using TCSPC into our automated platform. With the current instrumentation an entire 96-well microplate can be screened in around five hours (depending on how many images per well are captured and on sample brightness), which means that the imaging can be performed unsupervised overnight. In our strategy it is the aim to use the lifetime imaging as a high time resolution method for cross-checking our fast wide-field technique. However, the time-taken is in fact not prohibitively slow. Efforts are underway, from a data-analysis point of view, to incorporate Bayesian or maximum likelihood techniques [79] to allow the number of photons required to achieve a reliable fit to the data to be relaxed and as such it would be possible to speed up the acquisition of TCSPC data by at least a factor 3. Additionally highly sensitive detection technology has recently become available which significantly improves acquisition times for TCSPC-FLIM.

### Calculation of Acceptor Anisotropy

Processing of images from the wide-field component of the instrument was performed using bespoke software written in Matlab (The Mathworks, Natick, MA). The raw 1344×1024 pixel CCD images are saved in the Image Cytometry Standard (ICS), a format which is capable of storing multi-dimensional data, in any numeric format along with image history or meta-data. Importantly for our purposes, the 12-bit images acquired from the camera can be stored in the ICS image and the resulting anisotropy image calculated from these raw images can be saved as floating point numbers within the same image format. Keeping a consistent image format allows both raw and functional images to be displayed by the same image processing and viewing software packages (typically ImageJ and IrfanView which both support the ICS format).

Post acquisition the raw images are split into four separate sub-images corresponding to two wavelength channels in two orthogonal linear polarisation directions (an example of a pre-split image is given in Figure S4). Next the sub-images are registered in each wavelength channel. Two methods are available: the first is a manual routine where fiducial markers are identified in each image being registered and a projective 2-D transform is applied. The second method is an automated routine [80] which does not require user input, useful if obvious fiducial markers cannot be identified or a calibration image was not acquired. This routine is a rigid-body image registration performed using a cross-correlation technique which determines image rotation and pixel offset but not shear or scale (unnecessary in this case since the system does not suffer from significant chromatic aberration). A mutual information joint entropy matrix is computed for the input images and maximized using Powel’s direction set method [81]. The calculation can take a few minutes to execute but needs only be performed at the start of the image analysis process and needs only be run if the set-up of the microscope is changed by the user. The registration parameters are saved to a transformation file and can be called upon by the user when processing large batches of images recorded with the same microscope configuration. Since the reproducibility and reliability of the fluorescence anisotropy calculation is highly dependent on the signal-to-noise ratio in each pixel [82] the batch processing software allows a Gaussian convolution filter and masking routine to be implemented. A user definable rigid background subtraction, based on an intensity threshold, is also implemented in the software.

The fluorescence anisotropy for each voxel in each of the two wavelength channels is then calculated using equation (4).
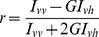
(4)Here *Ivv* and *Ivh* represent the fluorescence emission intensities measured parallel and perpendicular, respectively, for the case where the linear polarization of the excitation is oriented vertically. In a microscope set-up the differences in efficiencies in detecting light in orthogonal polarisation directions is accounted for using the G-factor shown in equation (5).



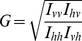
(5)


### Analysis of Fluorescence Lifetime Data

Here we give a brief summary of the processing and analysis of time-resolved images acquired by TCSPC using the modified Levenberg-Marquardt (MLM) algorithm [83] which has been implemented in a software package called Time Resolved Imaging (in its second major release called TRI2 available upon request from P Barber) [84]. The photon counts in each pixel of the image are binned by the ADC of the counting electronics and it is these photons that are used to determine the fluorescence lifetime in each pixel allowing the construction of a so-called time-resolved image. Although initially not designed with high-content screening in mind, the software provides the means to perform these calculations for large batches of images. The calculations can be done in a number of ways with the most recent release providing a means for performing global analysis on time-domain data [85]. The software attempts to fit a model similar to that given in equation (2) by iterative re-convolution (a mono-exponential fit is achieved by setting α_2_ = 0). The quality of the fit is judged using the reduced 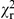 parameter, as given by equation (4). 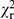 is minimised using the MLM algorithm. The key to the speed which the algorithm achieves is the rapid lifetime determination (RLD) method which is used to provide parameter estimates for the fitting; details can be found in [84], [86], [87]. The images can be processed on a pixel-by-pixel basis, where each image is treated as completely independent (for large batches the same initial parameters are applied to each image in turn) or the fitting can be done using global analysis where selected images, or indeed the entire dataset, can be fitted simultaneously.
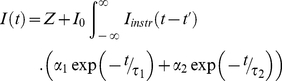
(6)

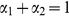
(7)

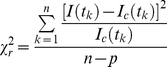
(8)In the above *I_0_* is the peak intensity; *α_1_* and *α_2_* are the fractional proportions of the lifetimes *τ_1_* and *τ_2_* respectively. *I_instr_(t)* is the instrument response function. *I(t_k_)* is the data and *I_c_(t_k_)* is the fit at time point *k*. *n* is the number of time points and *p* is the number of variable fit parameters. An example of lifetime data, and the fit generated, for a 5×5 group of pixels for an image of the 7AA FRET standard is given in Figure S5.

## Supporting Information

Figure S1 **Shows a plot of lifetime against anisotropy extracted on an image-by-image basis for each of the FRET standard constructs.** A calculation of Pearson’s coefficient shows the two methods of measuring FRET are highly correlated. If the GFP data is not included we calculate a coefficient of 0.75.
(TIF)

Figure S2 **A wide-field image of MTLn3E expressing CXCR4-eGFP and CXCR4-TagRFP after addition of CXCL12 for one hour and treatment with the dynamin inhibitor dynasore.** This image illustrates the origin of the lack of correlation between acceptor anisotropy and donor lifetime measurements. Dynasore is fluorescent and has a spectrally broad emission spectrum causing it to appear in both the eGFP and Tag-RFP channels. The appearance in the eGFP donor channel produces an average lifetime in these wells of around 0.7 ns. The wide-field image shown here illustrates that the dynasore is distributed throughout the glycerol mounting medium and this directly results an average anisotropy of around 0.39 in the acceptor channel.
(TIF)

Figure S3 **This montage shows twelve frames extracted from a live cell FLIM image sequence that was recorded over the course of twenty hours.** Each frame is separated by twenty minutes. The sequence shows the variation in Cdc42-Raichu activity. We see three clear different types of behaviour in this sequence. The red arrow indicates a cell which undergoes mitosis in the time-frame of the sequence. Initially Cdc42-Raichu activity is high (as indicated by the redder colours on this look-up-table) which then decreases when the two daughter cells spread and become part of the surrounding group of cells. The cell indicated by the white arrow is highly motile and has a correspondingly high Cdc42-Raichu activity. As the sequence proceeds the cell becomes less motile and eventually forms a junction with surrounding cells. This process correlates with a gradual reduction in the level of Cdc42-Raichu activity (as evidenced by the colours becoming bluer indicating an increase in fluorescence lifetime). Those cells that formed junctions before the sequence began remain in this state throughout the sequence and Cdc42-Raichu activity also remains low. (Scale bars represent 50 µm.)
(TIF)

Figure S4 **An example of a wide-field image which is divided into four spatially identical regions which differ in their wavelength and polarisation content.** The image is divided into two wavelength channels and two orthogonal polarisations.
(TIF)

Figure S5 **An example of a fit to donor fluorescence lifetime data using the software package TRI2.** The data is a 5×5 pixel region that has been extracted from an image of a Hela cell expressing a FRET standard with a 7AA linker. The data is fitted using the Levenburg-Marquardt algorithm within TRI2 using both a mono- and bi-exponential model. The residuals and χ^2^ values indicate the quality of the fit. There is a slight improvement when using a bi-exponential fit over the mono-exponential case but we chose to use the simpler model throughout since the fit has a χ^2^ value around 1.0. When constructing FLIM data the image is first processed using a block processing routine with a circular kernel and then each pixel in the 256×256 image is fitted as described here.
(TIF)
