# Supplementary figures and images for: A Multi-Functional Imaging Approach to High-Content Protein Interaction Screening
Source: PLoS One. 2012 Apr 10;7(4):e33231. doi: 10.1371/journal.pone.0033231 (PMC3323588; doi:10.1371/journal.pone.0033231)

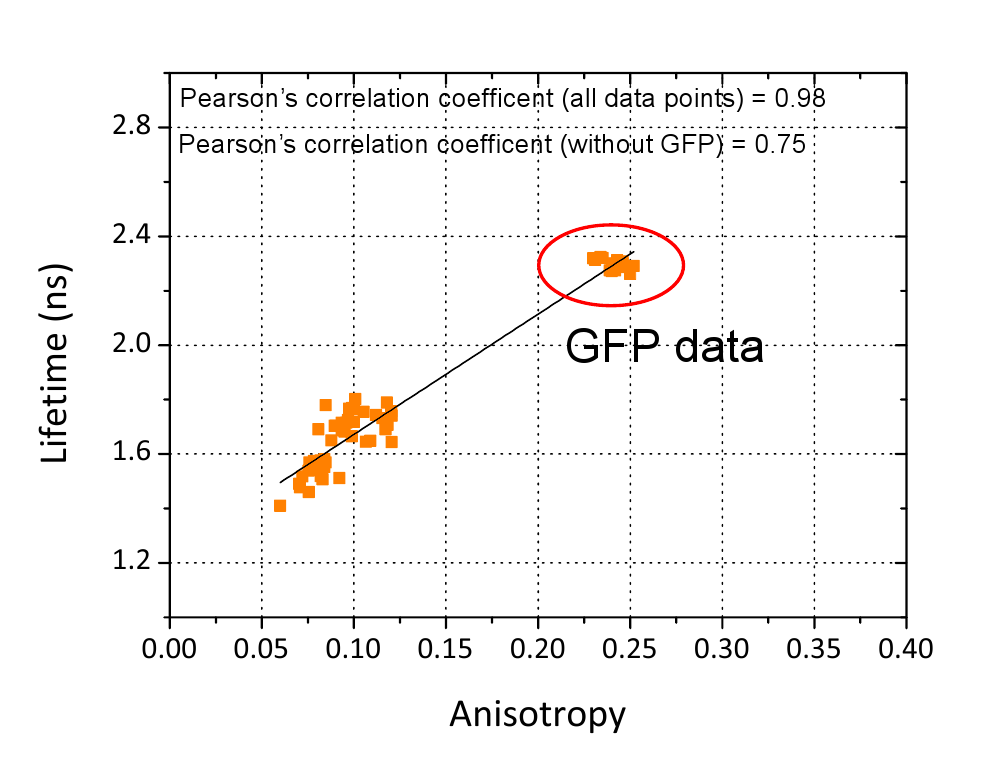

Supplement: Figure S1 — Shows a plot of lifetime against anisotropy extracted on an image-by-image basis for each of the FRET standard constructs. A calculation of Pearson’s coefficient shows the two methods of measuring FRET are highly correlated. If the GFP data is not included we calculate a coefficient of 0.75. (TIF) [file pone.0033231.s001.tif]

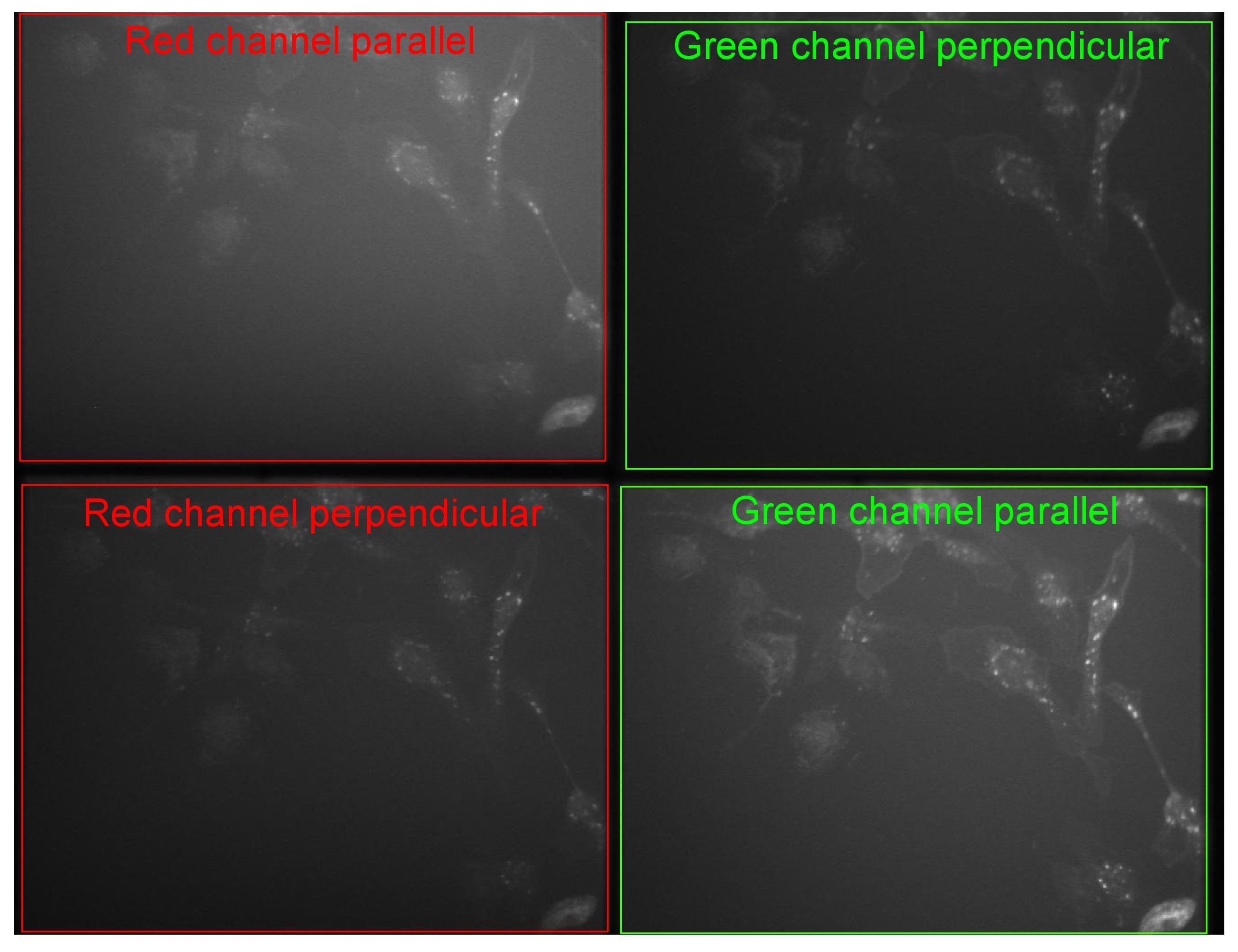

Supplement: Figure S2 — A wide-field image of MTLn3E expressing CXCR4-eGFP and CXCR4-TagRFP after addition of CXCL12 for one hour and treatment with the dynamin inhibitor dynasore. This image illustrates the origin of the lack of correlation between acceptor anisotropy and donor lifetime measurements. Dynasore is fluorescent and has a spectrally broad emission spectrum causing it to appear in both the eGFP and Tag-RFP channels. The appearance in the eGFP donor channel produces an average lifetime in these wells of around 0.7 ns. The wide-field image shown here illustrates that the dynasore is distributed throughout the glycerol mounting medium and this directly results an average anisotropy of around 0.39 in the acceptor channel. (TIF) [file pone.0033231.s002.tif]

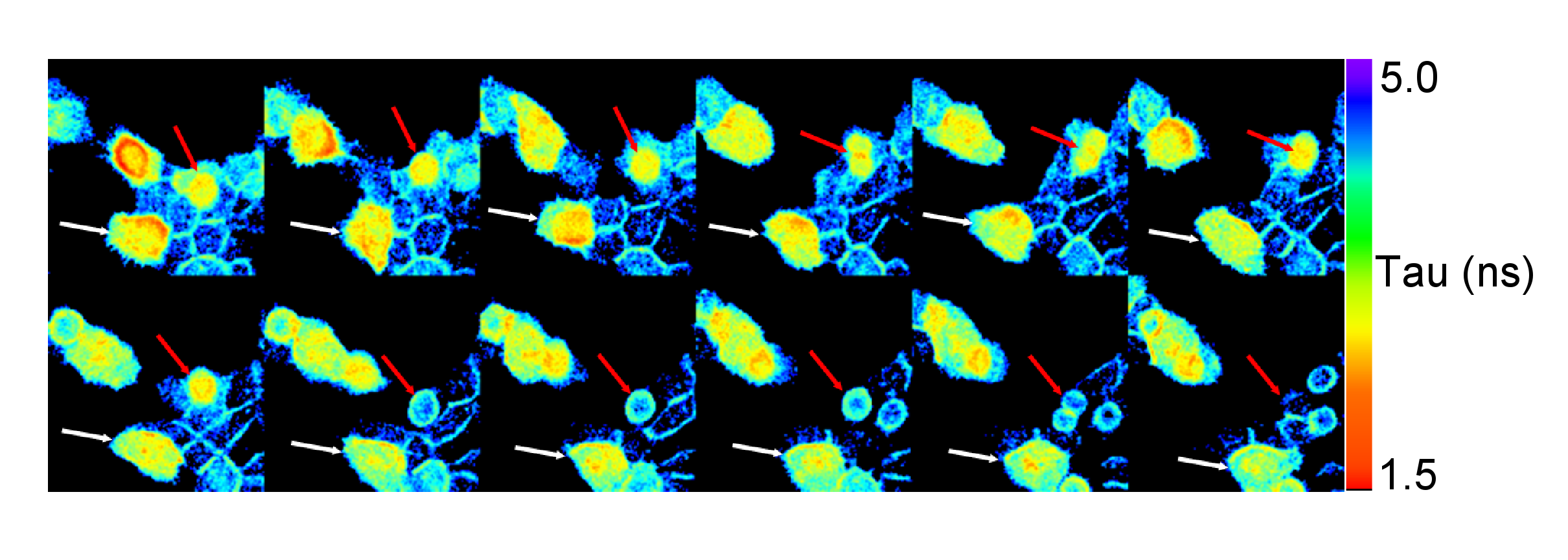

Supplement: Figure S3 — This montage shows twelve frames extracted from a live cell FLIM image sequence that was recorded over the course of twenty hours. Each frame is separated by twenty minutes. The sequence shows the variation in Cdc42-Raichu activity. We see three clear different types of behaviour in this sequence. The red arrow indicates a cell which undergoes mitosis in the time-frame of the sequence. Initially Cdc42-Raichu activity is high (as indicated by the redder colours on this look-up-table) which then decreases when the two daughter cells spread and become part of the surrounding group of cells. The cell indicated by the white arrow is highly motile and has a correspondingly high Cdc42-Raichu activity. As the sequence proceeds the cell becomes less motile and eventually forms a junction with surrounding cells. This process correlates with a gradual reduction in the level of Cdc42-Raichu activity (as evidenced by the colours becoming bluer indicating an increase in fluorescence lifetime). Those cells that formed junctions before the sequence began remain in this state throughout the sequence and Cdc42-Raichu activity also remains low. (Scale bars represent 50 µm.) (TIF) [file pone.0033231.s003.tif]

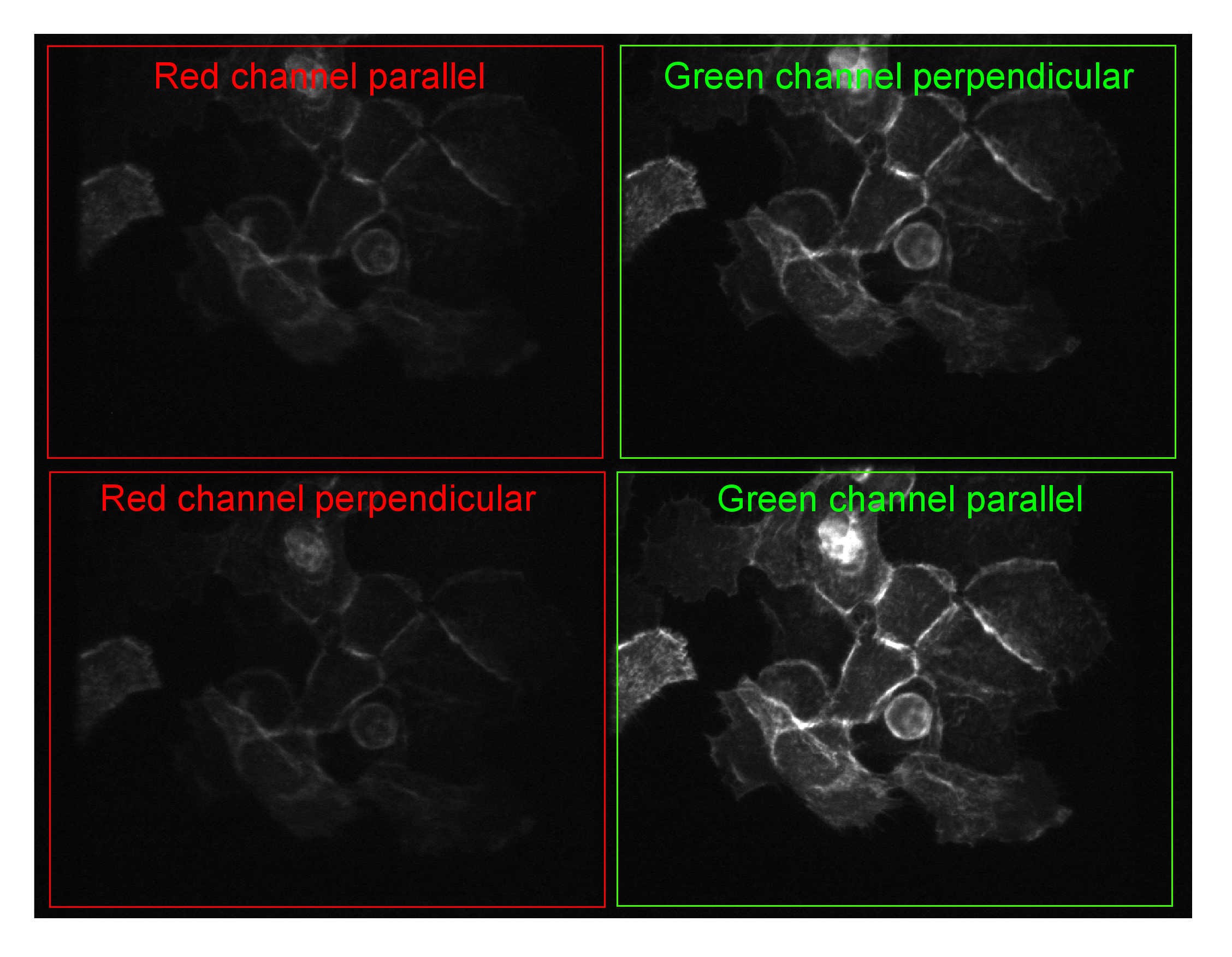

Supplement: Figure S4 — An example of a wide-field image which is divided into four spatially identical regions which differ in their wavelength and polarisation content. The image is divided into two wavelength channels and two orthogonal polarisations. (TIF) [file pone.0033231.s004.tif]

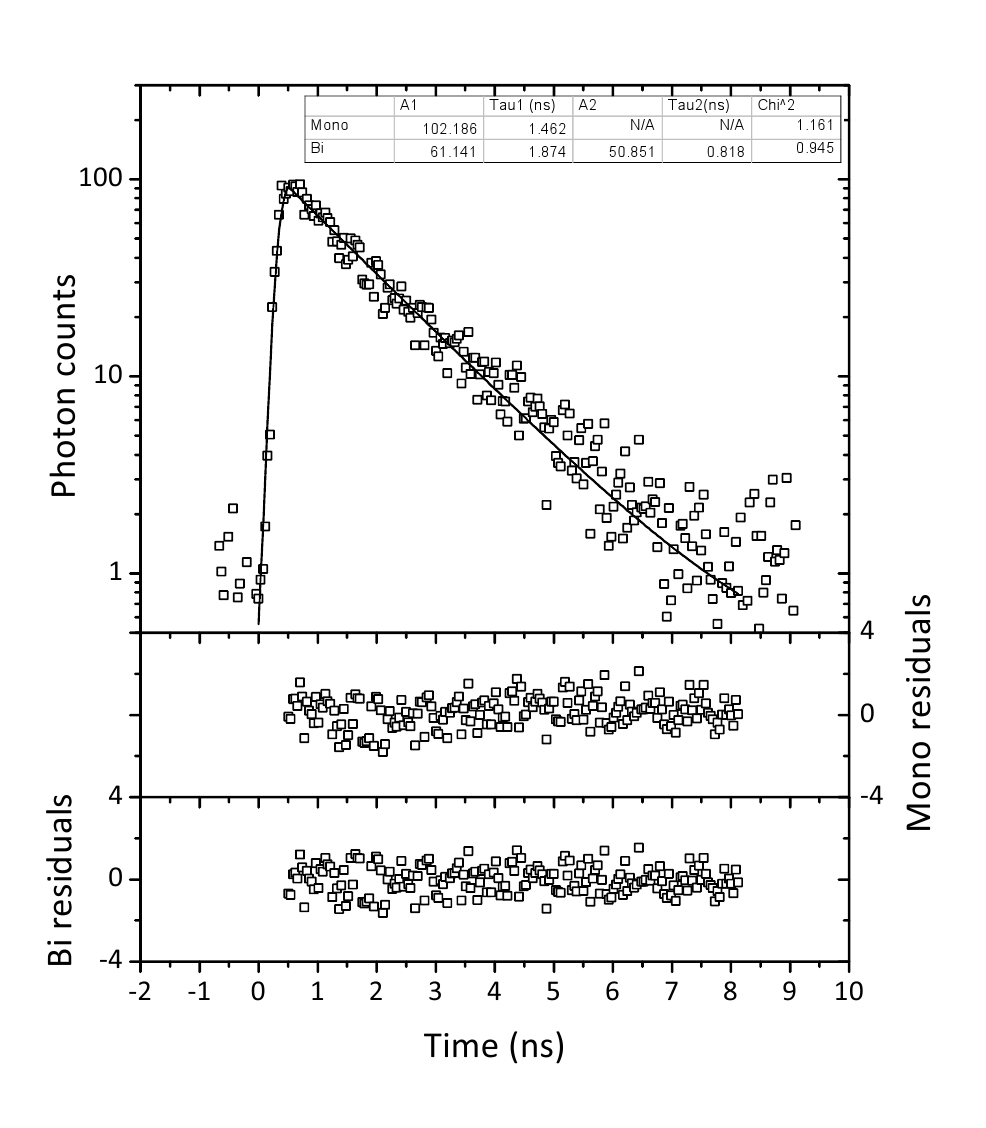

Supplement: Figure S5 — An example of a fit to donor fluorescence lifetime data using the software package TRI2. The data is a 5×5 pixel region that has been extracted from an image of a Hela cell expressing a FRET standard with a 7AA linker. The data is fitted using the Levenburg-Marquardt algorithm within TRI2 using both a mono- and bi-exponential model. The residuals and χ2 values indicate the quality of the fit. There is a slight improvement when using a bi-exponential fit over the mono-exponential case but we chose to use the simpler model throughout since the fit has a χ2 value around 1.0. When constructing FLIM data the image is first processed using a block processing routine with a circular kernel and then each pixel in the 256×256 image is fitted as described here. (TIF) [file pone.0033231.s005.tif]
